# Chronic low dose arsenic exposure preferentially perturbs mitotic phase of the cell cycle

**DOI:** 10.18632/genesandcancer.185

**Published:** 2019-02

**Authors:** Suthakar Ganapathy, Jian Liu, Rui Xiong, Tianqi Yu, Alexandros Makriyannis, Changyan Chen

**Affiliations:** ^1^ The Center for Drug Discovery, Northeastern University, Boston, MA, USA; ^2^ The First Affiliated Hospital of Nanchang University, Nanchang, Jiangxi Sheng, P.R. China

**Keywords:** Arsenic, mitosis, Plk1, BubR1, cyclin B1

## Abstract

Environmental pollution is a big challenge for human survival. Arsenic compounds are well-known biohazard, the exposure of which is closely linked to onsets of various human diseases, particularly cancers. Upon chronically exposing to arsenic compounds, genomic integrity is often disrupted, leading to tumor development. However, the underlying mechanisms by which chronic, low dose arsenic exposure targets genetic stability to initiate carcinogenesis still remain not fully understood. In this study, human lung epithelial BEAS-2B cells and keratinocytes were treated with 0.5 μM of sodium arsenite for one month (designated as BEAS-2B-SA cells or keratinocytes-SA), and its effect on cell cycle responses was analyzed. After being arrested in mitotic phase of the cell cycle by nocodazole treatment, BEAS-2B-SA cells or keratinocytes-SA were delayed to enter next cytokinesis. The lagging exit of the cells from mitosis was accompanied by a sustained Plk1 phosphorylation, which led to a persistent activation of the mitotic regulators BubR1 and Cdc27. As the result, cyclin B1 (clnB1) degradation was attenuated. BEAS-2B-SA cells or keratinocytes-SA also expressed a constitutively active Akt. The cytogenetic analysis showed an increased numbers of aneuploidy in these cells. The suppression of Akt reversed the aberrant expressions of the mitotic regulators, delay of mitotic exit as well as chromosomal aberrations. Our findings suggest that a long-term exposure to low dose sodium arsenite aberrantly retains the catenation of mitosis, which facilitates establishing genetic instability and predisposes the cells to tumorigenesis.

## INTRODUCTION

Arsenic compounds are toxic metalloids and exist in polluted water or air, especially in mining or industry areas, exposures of which are serious risks to human health [1, 2]. Epidemiological investigations indicated that chronic arsenic exposure is associated with the onsets of various human illnesses, in particular cancer [3-7]. Studies revealed that arsenic causes aberrant increases of reactive oxygen species (ROS) and further oxidative stress in cells [8, 9]. In arsenic-mediated carcinogenesis, PI3K/Akt and other mitogenic-related pathways were activated as well as p53 was suppressed [10-12]. Upon arsenic exposure, cytogenetic state of a cell was altered, leading to chromosomal aberrations and genetic instability [13-17]. On the contrary, it has also been reported that transient low dose arsenic treatment had the protective effect of normal tissues against damages caused by radiation- or chemo-therapy, which was through the metabolic switch from mitochondrial respiration to aerobic glycolysis [18, 19]. Despite these findings, the underlying mechanisms by which arsenic promotes tumorigenesis remain not fully understood. Because environmental arsenic pollution affects a large number of human beings worldwide, there is an urgent need for identifying intracellular targets of arsenic in its carcinogenic action, which will help developing new strategies for preventing or treating the diseases caused by the exposure to this metalloid toxin.

Cell cycle checkpoints function to ensure a tightly regulated cell cycle progression, which permits accurate assessing mitogenic signals, proper repairs of damages or efficiently preventing damaged genomes from being further propagated by eliminating severely damaged or unwanted cells [20, 21]. Checkpoint proteins are composed of cyclins, cell cycle-dependent kinases and phosphatases. During mitosis, centrosomes dictate a dynamic array of microtubules and establish bipolar spindles for duplicating/segregating chromosomes [22, 23]. The anaphase promoting complex (APC) functions as a multi-subunit E3 ubiquitin ligase and plays a crucial role in ensuring a proper mitotic progression and exit [24, 25]. APC-mediated cyclin B1 decay allows cells in mitotic phase to enter next cytokinesis [26, 27]. A failure of cells to exit from mitosis leads to the occurrence of genetic instability or mitotic catastrophe/death, depending upon cell types, stimuli or circumstances [28-33]. Arsenic compounds are mutagenic, which is through disrupting cell cycle restriction and chromosomal aberrations or deletions [17, 34]. It has been observed that human fibroblasts or cancerous cells aberrantly progressed in mitosis following arsenic treatment, which promoted formations of abnormal chromosomal structures or aneuploidies [17, 34, 35]. The inhibition of cyclin-dependent kinase 1 (Cdk1) that phosphorylates and keeps cln B1 in stable status was shown to partially reduce chromosomal aberration in arsenic-treated cells. Thus, it seems that arsenic-mediated cell cycle perturbation (especially disruption of mitosis) contributes to its carcinogenic activity.

Arsenic exposure changed the phosphorylation status of several mitogenic signal transducers and subsequently elicited protein kinase cascades [19, 36]. For example, Akt was activated after arsenic treatment, and further phosphorylated filamin A to enhance cell migration [19]. Polo-like kinase 1 (Plk1) was reported to be upregulated following the addition of high doses (> 5 μM) of arsenic, which either led to mitotic catastrophe or affected chromosomal maturation (such as chromosomal alignment or segregation) for perturbing genetic stability and promoting tumorigenesis [37-39]. The connection between Akt activation and Plk1 in response to arsenic treatment was also reported, which played a significant role in activating mTOR pathway for inducing the metabolic switch from oxidative phosphorylation to aerobic glycolysis in cells [19].

It was demonstrated that low doses of chronic arsenic exposure could induce aberrant chromosomal segregations, chromosomal instability and further tumorigenesis [17]. However, it is still unclear how the exposure affects the cell cycle restrictions, especially in mitosis, leading to aberrant chromosomal aberrations and further tumor promotion in mammalian cells. Therefore, in this study, we focused on investigating the effect of the chronic exposure of sodium arsenic on mitotic progression. We showed that normal cells chronically treated with the low dose sodium arsenite delayed to exit from mitosis. In this process, Plk1 was upregulated, accompanied with the persistent activation of BubR1 and Cdc27, leading to attenuate clnB1 degradation and chromosomal abnormality. The knockdown of Plk1 blocked the delay of mitotic exit. Furthermore, Akt is functioned upstream of Plk1 in this arsenic-mediated actions. Overall, our findings suggest that long-term arsenic exposure, *via* activating Akt, targets Plk1 to disrupt mitotic restriction, which potentiated genetic instability and tumorigenesis.

## RESULTS

### Low doses of sodium arsente treatment delay prolong cells to exit from mitosis

**Figure 1:**
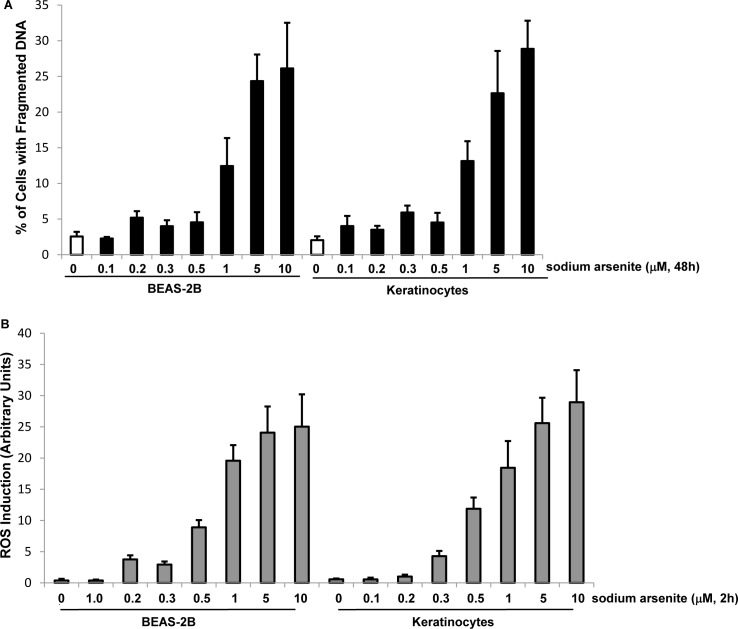
Responses of BEAE-2B cells or keratinocytes to different doses of sodium arsenite treatment **A.** Human lung epithelial BEAS-2B cells and keratinocytes were treated with different concentrations of sodium arsenite for 48 h and DNA fragmentation assay was then conducted to analyze the occurrence of apoptosis. **B.** Cells were treated with various doses of sodium arsenite for 2 h and stained with DCF to measure the levels of ROS. Error bars are the standard deviation (SD) over 5 experiments (n = 5; p < 0.05).

Studies showed that transient, low doses of arsenic treatment appeared to be beneficial for treatments of certain types of cancer, which could induce metabolic changes and inactivating p53 to avoid extensive normal tissue damages surrounding tumor lesions [10, 11, 18, 40]. However, the underlying mechanisms of chronic, low doses of arsenic exposure on tumor initiation remain not fully understood yet. To further investigating the mechanisms of this metal toxin, we tested the dose response of sodium arsenite in human lung epithelial BEAS-2B cells and keratinocytes to determine its sub-lethal doses. The cells were treated with various doses of sodium arsenite for 48 h and the induction of apoptosis was analyzed by DNA fragmentation assay (Figure 1A). BEAS-2B cells and keratinocytes started to become apoptotic at the concentration of 1.0 μM or higher of sodium arsenite. The magnitude of apoptosis was increased with increasing sodium arsenite concentrations.

Perturbation of the redox state in cells by arsenic exposure can significantly upregulate levels of reactive oxygen species (ROS), and further elicit oxidative stress that either damages cellular macromolecules (such as DNA, RNA, lipids and proteins) to promote tumorigenesis or induces apoptosis [41-43]. To determine sub-lethal doses of sodium arsenite, the levels of ROS in the cells treated with different concentrations of sodium arsenite for 2 h were measured (Figure 1B). The amounts of ROS in both cell lines were slightly increased after the treatment of sodium arsenite at 0.5 μM and significantly augmented with further increasing its concentrations. The data indicated that 0.5 μM of sodium arsenite affected redox state in the cells, which was not sufficient for triggering cell death. In addition, 0.5 μM of sodium arsenite is similar with that in contaminated environment [5-7, 36]. Therefore, this concentration of sodium arsenite was selected to be used in the following experiments.

**Figure 2:**
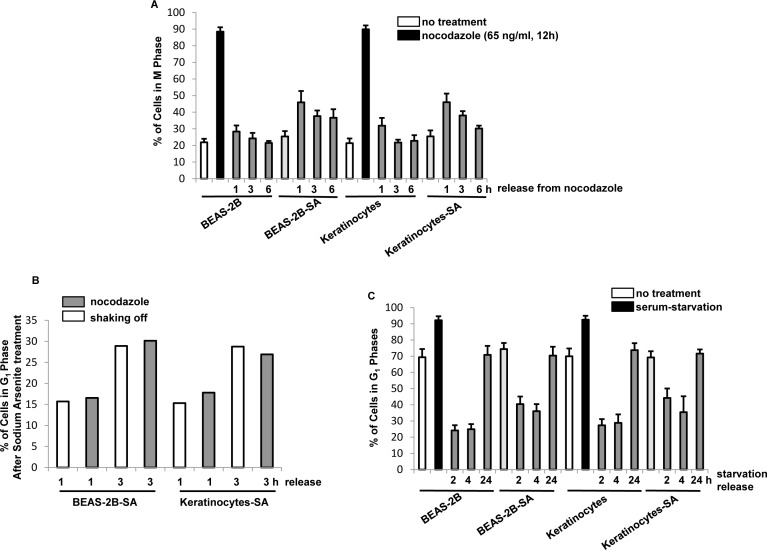
Mitotic accumulation in response to chronic, low dose sodium arsenite treatment **A.** After released from nocodazole block at different time points, the percentages of the cells in mitotic phase were measured by flow cytometry analysis. Error bars are the SD over 5 experiments (*n* = 5; *p* < 0.01). **B.** Mitotic cells were collected through mechanical shaking-off or by nocodazole block at different time points. After being re-propagated in the cultures for 1 or 3 h, the percentages of the cells in G_1_ phases were measured. Error bars are the SD over 5 experiments (*n* = 5; *p* < 0.05). **C.** Cells were cultured in the medium depriving the serum for 48 h and the percentages of G1 were measured by flow cytometry. Error bars are the SD over 5 experiments (*n* = 5; *p* < 0.01).

The exposure of arsenite compounds at high doses can disrupt cell cycle restriction and especially target mitosis, which damages the integrity of the genome and initiates tumorigenesis [36]. To test the influence of the chronic, low dose of arsenic exposure on mitotic phase, BEAS-2B cells and keratinocytes were treated with sodium arsenite (0.5 μM) for one month, which are designated as BEAS-2B-SA cells and keratinocytes-SA. After released from nocodazole block at different time points, the percentages of the cells in mitotic phase were measured by a flowcytometer (Figure 2A). In response to nocodazole treatment, approximately 90% of the cells with or without chronic, low dose of sodium arsenite treatment were accumulated in mitosis. After being released from nocodazole block, the most of the control cells rapidly entered into next cytokinesis. In contrast, chronic, BEAS-2B-SA cells and keratinocytes-SA delayed to exit from mitosis following nocodazole block was lifted. Even at 6 h of the releasing, more than 40% of BEAS-2B-SA cells and keratinocytes-SA still remained in mitotic phases.

To exclude the possibility that the lagged mitotic progression observed here might be due to the effect of nocodazole treatment, mitotic cells were collected through mechanical shaking-off or *via* nocodazole treatment. After being seeded, their exiting from mitotic phase was measured by the percentages of the cells residing in G_1_ phase 1 or 3 h later (Figure 2B). Comparable delays in mitotic exit were detected in the cells upon the different collections. Thus, the data confirmed that chronic, low dose arsenic exposure delayed the cells to exit from mitotic phase.

The effect of the chronic, sodium arsenite treatment on G_1_ phase of the cells cycle was also examined (Figure 2C). After released from serum-starvation at different time points, the percentages of the cells with or without chronic, low dose of sodium arsente treatment in G_1_ phase of the cell cycle were measured. In normal growth conditions, more than 70% of the control cells resided in G_1_ phase of the cell cycle, which increased to more than 90% in G_1_ phase in response to serum-starvation. Twenty-four hours later after being released from serum starvation, the majority of the cells (approximately 75%) with or without chronic, sodium arsenite treatment were detected in G_1_ phase. The data suggested that the low dose sodium arsenite exposure prolonged the duration of M phases of the cells, and did not affect G_1_ phase.

### Plk1 was activated in the cells chronically treated with low dose of sodium arsenite

**Figure 3:**
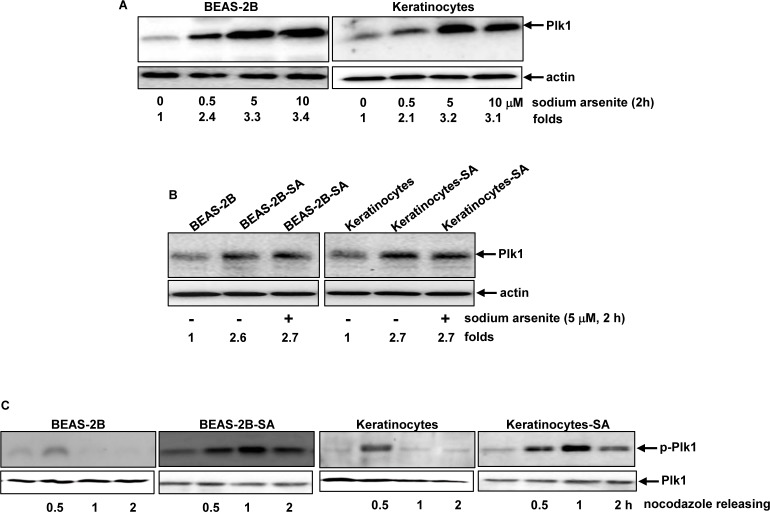
Plk1 activation in response to sodium arsenite treatment **A.** BEAS-2B cells and keratinocytes were treated with different doses of sodium arsenite for 6 h, and Plk expression was examined by immunoblotting analysis. Folds: increases of Plk1 induced by sodium arsenite treatment in comparison with that in untreated controld. **B.** Cells were stimulated with sodium arsenite for 6 h. Subsequently, the expression of Plk1 was analyzed by immunoblotting. Folds: increases of Plk1 induced by sodium arsenite treatment in comparison with that in untreated controls. **C.** After released from nocodazole block at different time points, the lysates from the cells were prepared and subjected to immunoblotting for the expression of the phosphorylated Plk1.

Polo-like kinase 1 (Plk1) plays important roles in regulating cell cycle-related activities, including centrosome maturation or bipolar spindle formation [44, 45]. The effect of the acute treatment of sodium arsenite at different doses on Plk1 expression in BEAS-2B and keratinocytes was examined (Figure 3A). The levels of Plk1 expression in both BEAS-2B cells and keratinocytes were moderately augmented in response to sodium arsenite treatment at 0.5 μM for 2 h, which was further increased by sodium arsenite at 5 μM and 10 μM with the similar increasing magnitudes. The expression of Plk1 was also analyzed in BEAS-2B-SA cells and keratinocytes-SA, with or without being re-stimulated with sodium arsenite (5 μM) for 2 h (Figure 3B). The moderate increase of Plk1 was detected in BEAS-2B-SA cells or keratinocytes-SA and however, the acute re-challenging of the cells with the high dose of this toxic metalloid (5 μM) did not further augment Plk1 expression.

The phosphorylation status of Plk1 was tested using immunoblotting analysis (Figure 3C). The phosphorylated Plk1 was detected in BEAS-2B-SA cells or keratinocytes-SA, which could not be recognized by the antibody in untreated BEAS-2B cells or keratinocytes. Half hour after released from nocodazole block, the increased levels of the phosphorylated Plk1 were present in all cells with or without chronically, low dose sodium arsenite exposure. However, the active form of Plk1 became undetectable in BEAS-2B and keratinocytes 1 h later of nocodasole releasing. In comparison, the phosphorylated Plk1 in BEAS-2B-SA cells or keratinocytes-SA remained to be seen at 2 h following the releasing. The data suggested that Plk1 phosphorylation or activation was sustained in the cells chronically treated with the low dose of sodium arsenite.

### Mitotic checkepoint was compromised after persistent, low dose of sodium arsenite treatment

**Figure 4:**
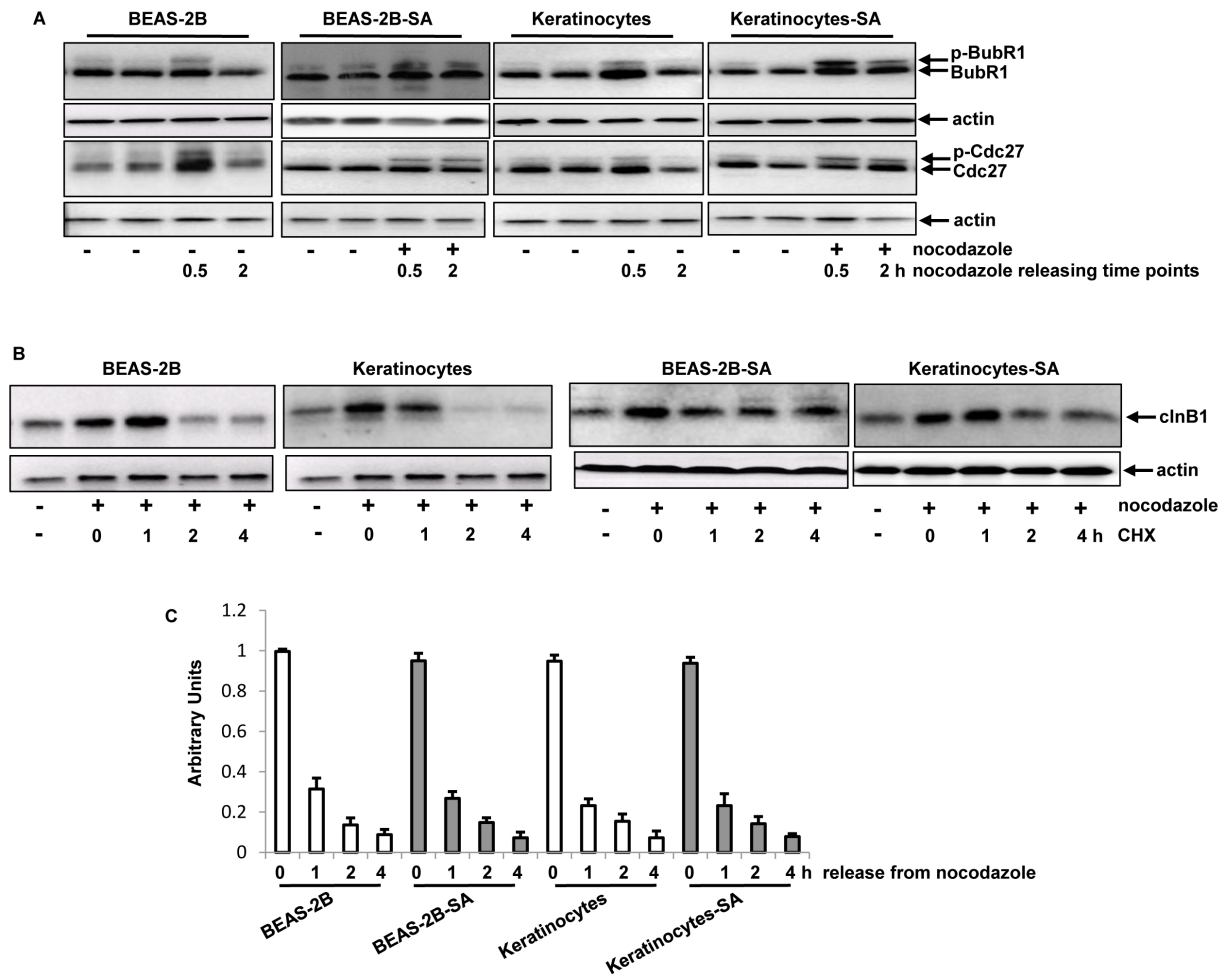
Effects of chronic, low dose sodium arsenite treatment on mitotic regulators **A.** The phosphorylated BubR1 and Cdc27 in the cells, after releasing from nocodazole block, were analyzed by immunoblotting analysis. **B.** After blocking protein synthesis by CHX, the levels of cln B1 expression in the cells were tested at different time points of nocodazole releasing. **C.** After adding actinomycin D (ATC) to block DNA synthesis, the cells were synchronized in mitosis by nocodazole treatment. Following releasing from mitosis block, quantitative PCR was performed to determine the levels of *^cln B1^* expression. Error bars represent SD from 5 experiments (*p* < 0.05).

Because Plk1 was activated in the cells chronically exposed to the low dose of sodium arsenite, the active statuses and durations of its downstream effectors (such as BubR1 and Cdc27) were examined. Anti-BubR1 and -Cdc27 antibodies that recognize the phosphorylated targets as slow-migrating bands and non-phosphorylated ones fast bands as fast-migrating bands on gels were used. Half hour of releasing from nocodazole treatment, the activated forms of these two mitotic regulators were detected, manifested as the presences of the slow migrating, phosphorylated BubR1 (p-BubR1) and Cdc27 (p-Cdc27) in the cells with or without chronic, low dose sodium arsenite treatment (Figure 4A). In comparison, 2 h after nocodazole release, the phosphorylated forms of BubR1 and Cdc27 were only detected in BEAS-2B-SA cells and keratinocytes-SA, but not in the control cells. These data presented the similar kinetics of the activations of Plk1, BubR1 and Cdc27 in the cells chronically treated with the low dose sodium arsenite, suggesting that BubR1 and Cdc27 might function downstream of Plk1.

Cyclin B1 (cln B1) is another mitotic regulator and, has to be degraded in order to allow cells to exit from mitosis and enter next cytokinesis [46]. Because chronic treatment of the low dose of sodium arsenite prolonged the activation of BubR1 and Cdc27, and caused mitotic accumulation, the stability of cyclin B1 in the cells was examined (Figure 4B). The cells were synchronized in mitotic phase by nocodazole prior to adding cycloheximide (CHX, a protein synthesis inhibitor). The levels of cln B1 in the cells at various time points following blocking protein synthesis were analyzed by immunoblotting analysis. Up to 2 h after the release from nocodazole block, cln B1 was degraded in the control cells. However, cln B1 in chronic sodium arsenite-treated cells, after the same treatments at the same time point, could still be detected, which lasted until 4 h later of CHX block.

In order to determine whether cln B1 stability in BEAS2B-SA cells or keratinocytes-SA was affected at the transcription level, quantitative PCR analysis was performed to test the kinetics of *cln B1* degradation (Figure 4C). After the co-treatment of nocodazole prior to adding actinomycin D (ATC, a transcription blocker), the levels of *cln B1* in the cells with or without the chronic, low dose of sodium arsenite treatment was reduced with similar kinetics. Thus, the data implicated that the delayed degradation process of cln B1 in the chronic sodium arsenite-treated cells was mainly affected at the protein level.

**Figure 5:**
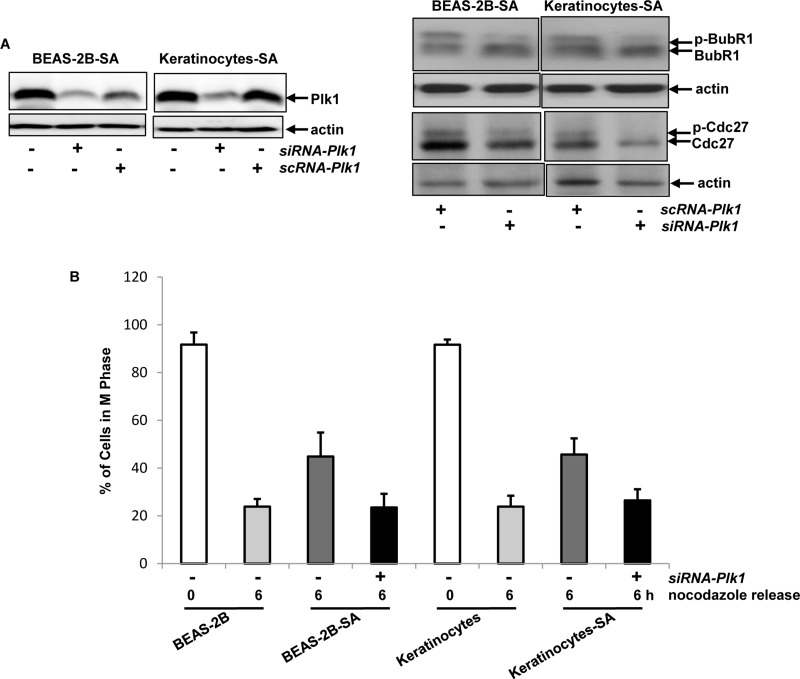
Effect of Plk1 on perturbing mitotic restriction induced by chronic, low dose sodium arsenite treatment **A.** After being treated with *sc* or *siRNA-Plk1*, the suppression of Plk1 expression in the cells was tested by immunoblotting (left panels). After the treatment of *scRNA-* or *siRNA-Plk1*, the expressions of the phosphorylated BubR1 and Cdc27 were examined in the cells that were released 1 h from nocodazole block (right panels). **B.** Six hours after released from nocodazole block, the percentages of the cells in mitotic phase were measured by flow cytometry. Error bars are the SD over 5 experiments (*n* = 5; *p* < 0.01).

### Plk1 was the target of arsenic exposure for perturbing M phase of the cells

Plk1 in BEAS-2B-SA cells or keratinocytes-SA was upregulated, accompanied by the activations of BubR1 and Cdc27. This led us to further determine the linear relationship among these mitotic regulators in our experimental setting. The *siRNA* was used to knock down *Plk1* in BEAS-2B-SA cells and keratinocytes-SA (Figure 5A, left panels). The treatment of *siRNA-Plk1*, but not the *scRNA* sufficiently knocked down more than two third of Plk1 in the cells. Subsequently, the phosphorylation of BubR1 or Cdc27 in the cells, after *Plk1* knockdown, was tested by immunoblotting analysis (Figure 5A, right panels). Two hour after nocodazole releasing, the phosphorylated form of BubR1 or Cdc27 in BEAS-2B-SA and keratinocytes-SA cells treated with *scRNA-Plk1* was detected by the antibody. After *Plk1* knockdown, the phosphorylated form of BubR1 or Cdc27 was no longer present. To further examine the effect of Plk1 on low dose sodium arsenite-mediated mitotic accumulation, BEAS-2B-SA cells and keratinocytes-SA were treated with *siRNA-Plk1* and then exposed to nocodazole. Six hours after released from nocodazole, the percentages of the cells in mitotic phase were analyzed (Figure 5B). After knockdown of *Plk1*, BEAS-2B-SA cells and keratinocytes-SA were able to rapidly exit from mitotic phase, the kinetic of which was similar as that of the controls. The results clearly suggested that Plk1 in BEAS-2B-SA cells and keratinocytes-SA functions upstream of BubR1, Cdc27 or clnB1 and they act in a hierarchy order to perturb the mitotic phase restriction.

### Akt acted upstream of Plk1 for disrupting mitotic restriction in chronic sodium arsenite treated cells

**Figure 6:**
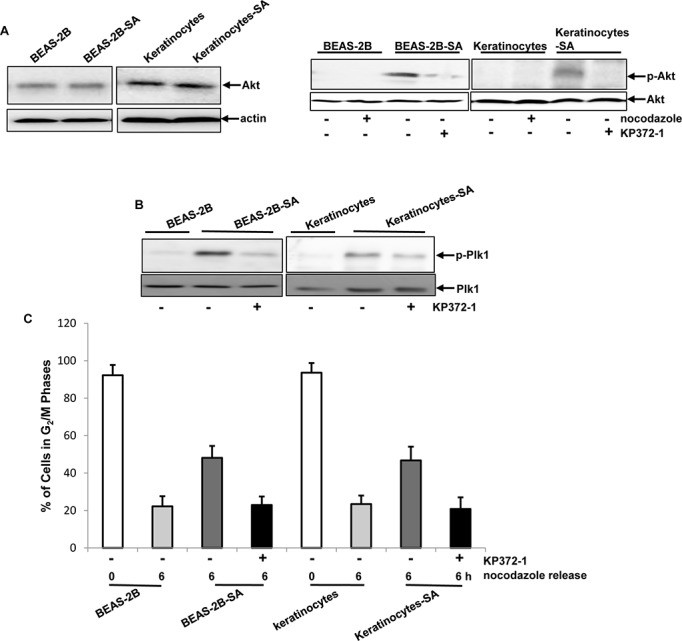
Importance of Akt in transmitting the chronic, low dose sodium arsenite-mediated signaling **A.** Levels of Akt expression in the cells was analyzed (left panels). The phosphophorylation status of Akt in the cells with or without the treatment of nocodazole or KP372-1 was analyzed (right panels). **B.** Plk1 phosphorylation was tested in the cells with or without KP372-1 treatment by immunoblotting. **C.** After the addition of KP372-1, the percentages of the cells in mitotic phase, 6 h following releasing from nocodazole block, were measured by flow cytometry. Error bars are the SD over 5 experiments (*n* = 5; *p* < 0.01).

The activation of Plk1 by arsenic treatment was suggested to link to PI3K/Akt or its related pathways [11, 19]. To determine whether Akt was proximal to chronic, low dose arsenic-induced signaling in our experimental setting, its expression was tested by immunoblotting analysis (Figure 6A, left panels). The levels of Akt expression in BEAS-2B-SA cells and keratinocytes-SA were similar as that of the control cells. Subsequently, its phosphorylation was analyzed (Figure 6A, right panels). The phosphorylated form of Akt was undetectable in the control cells in response to nocodazole treatment. However, the phosphorylated Akt was revealed by the antibody in BEAS-2B-SA cells and keratinocytes-SA, which was suppressed by adding KP372-1 (an Akt inhibitor).

It was shown that Akt and Plk1 functioned in concert to promote sodium arsenite-mediated actions [11, 12, 19]. To determine their relationship in our experimental setting, Plk1 activation after the inhibition of Akt was determined (Figure 6B). One hour after the release from nocodazole block, the phosphorylated form of Plk1 was seen in BEAS-2B-SA cells or keratinocytes-SA, as expected, which was suppressed by the addition of KP372-1. To further test the role of Akt in the sodium arsenite-mediated disruption of mitoitc phase, the cells with or without KP372-1 treatment were exposed to nocodazole. Six hours after releasing from nocodazole block, the percentages of the cells in mitotic phase was measured by a flow cytometer (Figure 6C). The inhibition of Akt activation by KP372-1 permitted BEAS-2B-SA cells or keratinocytes-SA to exit from mitosis within a proper time frame, the kinetics of which was similar as that of the control cells. Thus, the data suggested that chronic, low dose sodium arsenite treatment activated Akt, which might impose aberrant influences on Plk1 to perturb mitotic restriction and, further to potentiate the carcinogenic actions in normal lung or skin cells.

### Akt appeared crucial in triggering the disruption of genetic integrity by chronic exposure of the low dose of sodium arsenite

**Figure 7:**
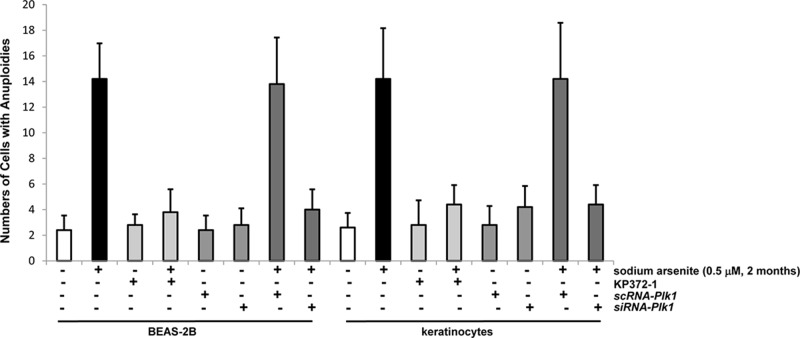
Chronic sodium arsenite treatment increases the frequency of aneuploidy in the cells The chromosomal spreads in the cells, after the inhibition of Akt or knockdown of Plk1, were prepared and then subjected to Giemsa staining. Frequencies of the cells with aneuploidy were quantitated in 100 cells per treatment, using a microscope. The measurements were plotted. Error bars are the SD over 5 experiments (n = 5; p < 0.05).

One of the consequences of deregulation of cell cycle restriction is the perturbation of chromosomal stability. In mitotic phase, aberrant chromosome segregations often lead to increasing frequencies of aneuploidy that is an indication of perturbed genomes [17, 34]. To explore if the disruption of mitotic restriction mediated by chronic, low dose arsenic exposure might link to the loss of genetic integrity, the rates of the occurrence of aneuploidy in the cells untreated or chronically treated with the low dose of sodium arsenite were measured (Figure 7). Because it takes longer time for establishing chromosomal abnormality, BEAS-2B cells and keratinocytes were cultured in the growth medium containing 0.5 μM alone as well as its co-treatment with KP372-1, *sc* or *siRNA-Plk1* for 2 months. Subsequently, the rates of aneuploidy in the cells were scored. An increase of aneuploidy was observed in BEAS-2B cells and keratinocytes after 2 months of the treatment with chronic, low dose of sodium arsenite alone. The suppression of Akt or Plk1 blocked the formation of aneuploidy induced by the chronic treatment of low dose sodium arsenite. The results clearly suggested that the perturbation of mitotic restriction indeed was an early and crucial factor for establishing genetic instability by this environmental metal toxin.

## DISCUSSION

Epidemiological studies and experimental research strongly indicate that chronic exposure to arsenic compounds through contaminated water or soil poses a great risk to human health worldwide [1, 2]. The persistent arsenic exposure at low doses causes various diseases, mainly cancer in the skin, lung or bladder among other organs. Such hazardous exposure induces cell cycle disruptions, mainly in mitosis, which is one of key elements for the establishment of chromosomal instability and further cancer development [28-32]. However, it is still not fully understood the molecular mechanisms by which chronic, low dose arsenitc exposure potentiates initiates tumorigenesis. For this reason, we conducted the investigation to explore how the low dose (0.5 μM) of sodium arsenite that is comparable with its concentrations in contaminated drinking water or soil dusts affects human lung epithelial BEAS-2B cells or keratinocytes. Our study demonstrated that after chronic exposure to the low dose of sodium arsenite, BEAS-2B cells or keratinocytes delayed to exit from mitosis. In this process, Plk1 activity was elicited and sustained, which was responsible for the prolonged activation of the mitotic regulators BubR1 and Cdc27, leading to strengthen clnB1 stability and subsequent mitotic accumulation of the cells. The experiments also showed that Akt functioned as the proximal effector of sodium arsenite-mediated signaling to connect the pro-growth signals to the nucleus. Thus, the results suggest that long-term, low dose exposure of sodium arsenite perturbs the mitotic restriction and prevents a proper exit of cells to next cytokinesis, leading to genetic instability of the cells and further potentiate cancer genesis and development.

The integrity of each phase of the cell cycle insures cells to timely progress or divide, as well as to properly repair damages. Arsenic exposure was able to aberrantly increase levels of ROS in cells, which caused DNA strand breaks, which preferentially affected mitotic phase and led to different consequences [12]. During chromosomal segregation in cells exposed to geno-stress, damaged or broken chromosomes cannot align closely to rejoining or being separated mechanically. DNA or chromosomal damage occurred in mitosis often induces a delay to prevent cells to exit from mitosis, which often induces chromosomal mis-segregations and generation of aneuploidy in daughter cells. For example, the treatment of taxol at low doses significantly augmented the numbers of mitotic cells, but microtubules polymer masses were not increased. As the result, chromosomal conformation was changed, which prevented tubulin dimerization from keeping the protofilament straight at the microtubule ends [47]. Sodium arsenite treatment at relative high doses (5 μM or above) was able to mimic mitotic poisons and induce mitotic accumulation by slowing down the rate of de-polymerization of the spindle. Subsequently, some cells surviving from high dose arsenic-induced injury acquired DNA or chromosomal alterations, and underwent transformation or tumorigenesis [48]. The molecular mechanisms of arsenic-mediated genetic instability are still needed to be fully explored.

Acute low dose arsenic treatment was shown to be cyto-protective for normal cells or tissues [11, 19, 40]. The treatment of low doses of arsenic induced a metabolic shift from oxidation to glycolysis, *via* blocking the functions of tumor suppressor p53, which reduced damages in normal cells or surrounding tissues caused by chemo-drugs or radiation therapy [11, 19]. The possible mechanism was that Plk1 phosphorylated Topors (topoisomerase 1-binding protein) to accelerate p53 degradation in response to low doses of arsenic exposure. Due to attenuating p53 function and switching to glycolysis, normal cells or tissues were given the growth and survival advantages for better recovery from the therapies tissues [11, 19, 40]. When switched glycolytic signaling was suppressed by depleting glucose supply, inhibiting hexokinase or suppressing lactate dehydrogenase, low dose arsenic treatment lost its protective function. Previously, we demonstrated that long-term, low dose of sodium arsenite treatment disrupted p53 function and induced ER stress in human lung and skin cells, which promoted transformation process [12]. In current study, we further showed that chronic, low dose arsenic treatment, *via* activating Akt, upregulated Plk1 activity, which perturbed mitotic restriction of the lung epithelial cells or keratinocytes. Together, these studies indicate that both acute and chronic, low dose of arsenic exposures are able to suppress p53 and upregulate Plk1 function for achieving either protective or carcinogenic outcome, respectively. It is unclear whether the transient suppression of p53 and change of metabolic pattern induced by transient, low dose arsenic exposure may have a profound impact on normal cells or tissues later on, with respect of tumor promotion. Such consideration should be rendered for the clinic uses of arsenic-based drugs.

Mitosis is a complicated process that involves four distinct sub-phases and, the transition from each sub-phase to the next one is timely controlled by the expressions of cyclins and activities of cell cycle kinases (CDKs) or phosphatases. Cln B1 and CDK1 are key factors for governing cells to enter into and progress through the early phages of mitosis. At the late stage of mitosis, cln B1 is dephosphorylated by the phosphatase of the anaphase promoting complex (APC) and subjected to be degraded, which allows cells to exit from mitosis and enter into next cytokinesis. Our study revealed that chronic, low dose arsenite treatment prolonged mitotic phase by increasing clnB1 stability, but it did not alter the length of a cell cycle of the cells. As the result, the frequency of aneuploidy in the cells chronically exposed to the low dose of sodium arsentie was increased, which links the perturbation of mitosis to arsenic exposure-mediated carcinogenesis.

Taken together, our study indicates that chronic, low dose arsenic treatment timely perturbs the length of mitosis in normal lung and skin cells. The consequences of the disruption seem preventing proper chromosomal segregations, and facilitating the establishment of chromosomal instability, leading to tumor initiation and development. This timely disruption of mitosis by the exposure to chronic, low dose sodium arsenite requires the collaboration of multiple effectors, such as Akt, Plk1 and its downstream effectors. Since arsenic exposure is one of the top environmental contaminant concerns for human health, the identification of molecular targets of arsenic-mediated carcinogenesis will help for the development of new strategies for better environmental protection as well as for designing new therapeutics to treat arsenic exposure-induced human malignancies.

## MATERIALS AND METHODS

### Cells culture and treatments

Human lung epithelial BEAS-2B cells were purchased from ATCC (Manassas, VA) and skin keratinocytes were generous gift from Dr. Lou (Boston University School of Medicine, MA). The cells were cultured in RPMI 1640 supplemented with 10% heat-inactivated fetal bovine serum (Invitrogen, CA) at 37°C in a 5% CO_2_ humidified atmosphere. Prometaphase arrest was achieved by treating cells with 65 ng/ml of nocodazole for 12 h. In general, more than 90% of cells were in mitosis. For collecting G_1_ population, cells were grown into monolayer and then in the medium containing 1% of serum for 48 h.

### Reagents and antibodies

Sodium arsenite (Sigma, MO) were administered from stock solutions in DMSO. The anti-phor-Akt, -Akt, phor-Plk, -Plk and -cln B1 antibodies were from Cell Signaling Technology (Danvers, MA). Anti-BubR1 antibody was a generous gift from Dr. Dai (New York University). Cdc27 antibody was from Rockland Immunochemicals Inc. (Limerick, PA). The antibody against Plk1 was from Santa Cruz Biotechnology, Inc. (Santa Cruz, CA).

### *In vitro* transfection with siRNA

Plk1 SMARTpool siRNAs were purchased from Dharmacon (Lafayette, CO), which contained 4 selectively designed siRNA with “UU” 3′-overhangs and a 5′-phosphate on the antisense strand. The cells were transfected with Plk1 SMARTpool siRNAs (siPlk1) or a nonspecific pooled control siRNA (siPC) (Dharmacon), using the LipofectAMINE 2000 (Invitrogen, Carlsbad, CA) reverse transfection protocol, according to the manufacturer's instructions.

### DNA fragmentation and cell cycle assays

Flow cytometric analysis was performed with a FACScan machine (Becton Dickenson, Mountain View, CA). The data analysis and display were performed with the Cell-Fit software program (Becton Dickenson). Cell-Fit provides data from the flow cytometer and real-time statistical analysis of the data. After various treatments, cells (1 × 10^6^/ml) were washed with 1 x PBS, fixed with 70% ethanol. Subsequently, cells were stained with propidium iodide (0.1 ug/ml) containing RNase at 1.5 ng/ml. The stained samples were kept at 4°C overnight before flow cytometric analysis for either measuring percentages of less G_1_ DNA contents of apoptotic cells or DNA profiles of cells in different phases of the cell cycle.

### ROS analysis

Treated or untreated cells were washed with ice-cold PBS and resuspended in 5 μg/ml of 2′, 7′-dichlorodihydrofluorescein diacetate (DCF) (Thermo Fisher Scientific, MA). The samples were incubated for 10 min at room temperature and analyzed immediately.

### Immunoblotting analysis

Cell lysates were extracted using a 1% SDS lysis buffer. DNA was removed by centrifugation at 13,000 rpm at 4°C for 100 min. Protein concentration was determined by measuring the absorbance at 585nm of proteins in a Bradford assay (Sigma, MO). Lysates were run on 12% of SDS-PAGE gels. Subsequently, membranes were blocked in 5% non-fat milk in TBS-tween and probed with corresponding antibodies. Blots were stripped and re-probed for β-actin as a loading control.

### Quantitative real time-PCR

Total RNA was isolated using TRIzol reagent (Invitrogen) according to the instruction provided by the manufacturers. cDNA was prepared using 1 μg of total RNA extracted (iScript cDNA synthesis kit, Bio-Rad), and then subjected for the amplification by quantitative real time-PCR using Applied Biosystem StepOnePlus in the presence of SYBR Green JumpStart (Sigma-Aldrich). Ribosomal 18 S RNA was used as the normalization control. The n-fold change in mRNA expression was then determined. The human *clnE* sense primer is: 5′-gtcctggctgaatgtatacatgc-3′ and the antisense primer is: 5′-cctatttgttcagacaacatggc-3′.

### Scoring aneuploidy

Untreated cells or chronic sodium arsenite (0.5 μM, 2 months) treated cells with or without other treatments were grown to reach > 80% and colcemid (0.1 μg/ml) was added into the cultures for 60 min. Metaphase cells were harvested by vigorously shaking. Cells were then suspended in hypotonic buffer at 37°C and fixed in cold ethanol: acetic acid (3:1). Fixed cells were dropped on slides that were heated at 88°C in 1 M NaH_2_PO_4_ (pH 8.0). The slides were stained with Giemsa for 5 min. For each treatment, chromosomes from 100 cells were scored for aneuloidy under a microscope.

### Statistical analysis

Statistical analysis was performed using a two-tailed Student's *t* test for comparison of two groups or a one-way analysis of variance for comparison of more than two groups followed by Tukey's multiple comparison tests. Tumor-free probabilities were estimated using Kaplan-Meier method and were compared among groups. Standard deviations are displayed in the figures. A p value < 0.05 was considered significant.
